# Social marketing-based interventions to promote healthy nutrition behaviors: a systematic review protocol

**DOI:** 10.1186/s13643-021-01625-5

**Published:** 2021-03-11

**Authors:** Azam Doustmohammadian, Marjan Bazhan

**Affiliations:** 1 Gastrointestinal and liver Diseases Research Center, Iran University of Medical Sciences, Tehran, Iran; 2 Department of Community Nutrition, Faculty of Nutrition Sciences and Food Technology, National Nutrition and Food Technology Research Institute, Shahid Beheshti University of Medical Sciences, #7, Arghavan St, Farahzadi Blvd, Shahrak Gharb, Tehran, Iran

**Keywords:** Healthy nutrition behavior, Social marketing, Intervention, Systematic review

## Abstract

**Background:**

Diet-related non-communicable diseases (NCDs) are rapidly increasing worldwide and constitute one of the leading causes of mortality and morbidity. Improving population diets can play an important role in preventing and managing the diseases. Effective and efficient interventions are needed to promote healthy eating behaviors among people. The objective of this review will be to evaluate the effectiveness of social marketing-based interventions to promote healthy nutrition behaviors.

**Method:**

The following electronic databases will be searched from January 1990 onwards: PubMed/MEDLINE, EMBASE, Web of Science, and CENTRAL. We will include randomized and non-randomized trials, quasi-experimental studies, observational studies (e.g., cohort, cross-sectional, and before and after studies) evaluating the social marketing-based intervention.

The primary outcomes will be nutritional behaviors. Secondary outcomes will include the quality of life, nutritional status, and weight status. Two reviewers will independently screen all citations, full-text articles, and abstract data. The study methodological quality (or bias) will be appraised using an appropriate tool. If feasible, we will conduct random-effects meta-analysis. Additional analyses will be conducted to explore the potential sources of heterogeneity (e.g., age, sex, and socio-economic condition).

**Discussion:**

This study will summarize the evidence regarding the interventions’ components, implementation methods, and effectiveness of interventions based on the social marketing framework to promote healthy nutrition behaviors. This review can provide policymakers with the information needed to make decisions and plan to promote healthy eating behaviors and understand the factors influencing the implementation of these programs.

**Systematic review registration:**

CRD42020163972

**Supplementary Information:**

The online version contains supplementary material available at 10.1186/s13643-021-01625-5.

## Background

The growing burden of non-communicable diseases (NCDs) is one of the biggest challenges facing healthcare systems worldwide in the twentyfirst century [1]. NCDs have been identified as major causes of disability and mortality globally. During 2016, about 71.3% of all deaths worldwide were claimed to be due to these diseases [2]. It has been projected that by 2030, NCDs will cause over 75% of all deaths worldwide [3]. Evidence suggests that NCDs are among the main leading causes of death in Iran. According to the latest statistics provided by the World Health Organization, NCDs are responsible for more than 76% of all deaths in Iran. Among these, coronary heart diseases make the most considerable contribution to NCD deaths (by 46%) [4]. The four modifiable behavioral risk factors for NCDs are physical inactivity, unhealthy diet, harmful alcohol use, and tobacco use. It is noteworthy that the NCD-related morbidity and mortality burden due to an unhealthy diet is much more significant than the other three risk factors [5].

During the last four decades, significant changes have taken place in global nutrition patterns. People’s diets have shifted to higher consumption of processed and ultra-processed foods low in nutrients and high in calories [6, 7]. The percentage of foods consumed outside the home, such as fast food, has increased, leading to excessive calorie intake [8]. Besides, there have been significant increases in the consumption of animal-source foods, oils, and caloric sweeteners [9]. In many countries, intakes of sodium, unhealthy fats, and added sugar in the general population are much higher than the recommended amounts [10]. It is important to note that increased consumption of unhealthy foods and beverages has been associated with a lower intake of healthy diet components, including foods with low-energy density and a high content of nutrients such as vegetables, legumes, and whole grains [9]. These dietary shifts have important implications for public health. Unhealthy eating patterns, including high consumption of added sugar, trans fats, and sodium, have been associated with obesity, cardiovascular disease, type 2 diabetes, cancer, high blood pressure, and stroke [11–13]. Also, such eating patterns have been reported to kill more than 14 million people worldwide each year, equivalent to 40% of deaths due to NCDs [14].

According to the World Health Organization, a healthy diet includes achieving energy balance, limiting energy intake from total fats, replacing saturated fats with unsaturated fats, eliminating trans fatty acids, limiting the intake of free sugars, limiting salt (sodium) consumption from all sources, and increasing consumption of fruits and vegetables, and legumes, whole grains, and nuts [15]. Many efforts have been made to develop nutritional interventions around the world. These primary prevention programs focus on asymptomatic individuals in the general population before an adverse health event occurs [16]. The goal of interventions at this stage is to reduce non-communicable disease risk factors by promoting healthy diets. There are powerful potential interventions that target the factors mentioned above and the cost, access to healthy foods, and individuals’ knowledge. It should be noted that such population interventions, by their nature, should be theoretically useful for all members of society, including those with a history of non-communicable diseases such as cardiovascular disease [17]. However, given the poor quality of the average diet, it is clear that current approaches to improve public nutrition have not been successful and that effective strategies are urgently needed [18].

Health-related behaviors, including nutritional behaviors, are influenced by a wide variety of factors. Understanding the multifactorial nature of these behaviors and the contexts in which they occur is essential for developing effective behavior change interventions. Having a clear theoretical underpinning has been suggested to maximize behavior change interventions [19–21]. Evidence shows that theory-based interventions effectively influence health-related behaviors than non-theory-based ones [22–24]. These types of studies provide a framework for developing and evaluating interventions [25]. They also facilitate understanding the factors influencing behavior change and the reasons for an intervention’s success or failure [26, 27]. One of the behavioral theories that is widely used to influence health behavior is social marketing.

There are many definitions of social marketing, but one of the most useful ones describes social marketing as follows: “Social marketing is the application of commercial marketing technologies to analyze, plan, implement, and evaluate programs designed to influence the voluntary behavior of the target audiences to improve their personal and social well-being” [28]. The unique feature of social marketing is that it takes learning from the commercial sector and applies it to induce, encourage, and promote social change entirely, rather than to provide ideas or information [29]. The strengths of social marketing over alternative techniques applied to behavior change include (1) focusing on the consumer—this means that interventions are designed based on the consumers’ wants and needs, not only the priorities of society as a whole [30]; (2) proposing attractive exchanges for the consumer, which makes it possible to change voluntary behavior; in other words, it focuses on convincing not on coercion [31], (3) identifying and appropriately addressing competition to make the recommended products or health behaviors more advantageous than the unhealthy ones [32], and (4) using all the marketing mix components (the basic 4 Ps: product, price, place, and promotion). In social marketing, a product is developed (key benefits, service, or behavior change), the price is evaluated (the costs of stopping an unhealthy behavior or adopting a healthy one, which can be in the form of money, time, opportunity, energy, effort, psychological factors, etc.), the place is defined (the situations in which the target audience can perform the health behavior or obtain any tangible goods or services necessary for the health behavior), and a promotion strategy is planned (selecting promotion techniques and channels) [33], (5) managing programs properly through planning, control, monitoring, implementation, and evaluation [28]. Accordingly, social marketing can be an excellent tool for promoting public health activities.

It is not a new idea to use marketing principles in health education and health promotion to achieve health or social goals [34–36]. Social marketing has been applied to solve behavioral problems for more than 40 years [37]. There are many examples of effective use of social marketing framework in the field of public health, including promoting healthy eating behaviors [38], prevention and control of AIDS [39], reducing alcohol consumption in youth and adolescents [40], and increasing physical activity [41]. Given the effectiveness of social marketing in changing health behaviors, the present review intends to collect interventional studies that have used the social marketing framework to promote healthy eating behaviors. The objective of this review will be to evaluate the effectiveness of social marketing-based interventions to promote healthy nutrition behaviors.

## Methods

### Protocol and registration

The present protocol was registered in the PROSPERO database under registration number CRD42020163972 and is being reported according to guidance provided in the Preferred Reporting Items for Systematic Reviews and Meta-Analyses Protocols (PRISMA-P) statement [42], (see Additional file 1). The proposed systematic review will be conducted based on the methodological guidance from the Cochrane Collaboration Handbook of Systematic Reviews [43] and will be reported following the Preferred Reporting Items for Systematic Review and Meta-Analysis (PRISMA) Statement [44]. Relevant changes to the protocol will be documented and published within the results of the final review.

### Eligibility criteria

#### Types of studies

We will include different types of study designs, including randomized-controlled trials (including cluster trials), non-randomized controlled trials, quasi-experimental studies, and observational studies (including cohort studies, cross-sectional studies, and before-after studies). Pre-and post-interventions are also common and will help determine the effect (if any) of interventions within a single community. The controlled cohort studies and cross-sectional studies will help identify differences in the communities’ outcomes, either overtime or within a particular point in time, respectively.

#### Types of participants

All population groups, including children, adolescents, adults, or older adults, and the community in different settings (kindergarten, school, home, or community), will be examined.

#### Types of interventions

The following interventions will be included in the study:

Any intervention to improve eating behaviors including increasing fruit and vegetable, legumes, whole grains, and nuts intake, healthy and low-fat choices (e.g., snack or meal alternatives with lower fat, energy, salt, sugar or higher fiber), other behaviors including variety, novel foods, fiber intake, low-sodium intake, consuming healthy breakfasts, healthy school lunches, and the emphasis on daily water consumption [45–52].

Studies must be social marketing-based interventions to promote healthy nutrition behaviors. The interventions that consider all the marketing mix components to improve nutritional behaviors will be included. It consists of the basic 4 Ps of product, price, place, and promotion. Product refers to healthy eating behaviors. Price refers to the cost for the promised benefits or the barriers that may prevent the people from performing healthy eating behaviors and what methods used in interventions to remove these barriers. Place refers to the location where people can obtain information about healthy eating behaviors, and finally, promotion includes the type of persuasive communication to improve eating behaviors.

The evidence from qualitative research will also be used to explain the effect of social marketing-based interventions on nutrition behaviors.

#### Types of outcome measures

Studies will be included in the review if they have quantifiable measures of the primary outcome. The primary outcomes will be nutritional behaviors including the following:

- Increased daily consumption of fruit, vegetables, legumes (e.g., lentils and beans), nuts, and whole grains servings [45].
- Healthy choices, including snack or meal alternatives with lower fat, energy, salt, sugar, or higher fiber. Less than 5 g of salt (equivalent to about one teaspoon) per day [46].
- Improvement in dietary intake indicators (e.g., Dietary Diversity Score (DDS) and Healthy Eating Index (HEI), diet quality indices (DQI), and Healthy Dietary Habits Index (HDHI)) [53, 54].
- Prefer unsaturated fats (found in fish, avocado and nuts, and in sunflower, soybean, canola, and olive oils) to saturated fats (found in fatty meat, butter, palm and coconut oil, cream, cheese, ghee, and lard) and trans-fats of all kinds, including both industrially produced trans-fats (found in baked and fried foods, and pre-packaged snacks and foods, such as frozen pizza, pies, cookies, biscuits, wafers, and cooking oils and spreads) and ruminant trans-fats (found in meat and dairy foods from ruminant animals, such as cows, sheep, goats, and camels).
- Reduced intake of saturated fats to less than 10% of total energy intake and trans-fats to less than 1% of total energy intake [49].
- Reduced intake of industrially produced trans-fats [50, 51].
- Less than 10% of total energy intake from free sugars [45, 52]. Free sugars are all sugars added to foods or drinks by the manufacturer, cook, consumer, and sugars naturally present in honey, syrups, fruit juices, and fruit juice concentrates.
- Other behaviors, including variety, novel foods, consuming healthy breakfasts, healthy school lunches, and the emphasis on daily water consumption [45].

Secondary outcomes will include improvement in the quality of life, nutritional status, and weight status.

Only articles published in English will be included. We will exclude reviews, methodological articles, and conceptual documents (e.g., non-empirical studies). We will exclude studies without a control group, not reporting outcome data, and/or addressing diseased/medically diagnosed populations.

### Information sources and search strategy

The primary source of literature will be a structured search of major electronic databases (from January 1990 onwards): PubMed/MEDLINE, EMBASE, Web of Science, and the Cochrane Central Register of Controlled Trials (CENTRAL). The secondary source of potentially relevant material will be a search of the grey or difficult to locate literature, including ProQuest and Google Scholar. We will perform hand-searching of the reference lists of included studies, relevant reviews, or other relevant documents. Authors who are prolific in the field will be contacted. The literature searches will be designed and conducted by the review team, including an experienced health information specialist. The search will include a broad range of terms and keywords related to nutrition behavior, social marketing, and interventions. A draft search strategy for the main databases is provided in Additional file 2.

### Data management and selection process

All the identified studies of different sources will be transferred to Endnote and systematically de-duplicated through The Systematic Review Assistant-Deduplication Module (SRA-DM). This freely available application has greater sensitivity and specificity than Endnote for the de-duplication process [55]. The remaining studies will be screened for eligibility by two independent reviewers. The resolution of any conflicts will be made by a third independent reviewer or by discussion. The reasons for the exclusion of any article will be documented. A full-text screening will then be carried out. A PRISMA flow chart showing details of studies included and excluded at each stage of the study selection process will be provided [56].

### Data extraction

We will extract data from the included studies using the pre-standardized data extraction form (see Additional file 3). The form will be tested and adapted as necessary before using it. Data extraction form will capture the following data fields: study title, author(s), publication year, geographic origin, study design, enrolment period, sample size, duration of intervention, participant characteristics (age, sex), intervention type, and its components, primary and secondary outcomes. Two authors will independently extract the data. We will discuss any differences between the two data extraction sheets to reach a consensus or consult a third author if an agreement is impossible to achieve. We will also contact study authors to obtain any missing information or clarify unclear data by getting the original report.

### Assessment of risk of bias in included studies

To estimate the potential bias that will be most relevant for the study, we will use the following tools: the Cochrane tool for assessing the risk of bias in randomized trials (RoB 2 tool) [57], the Risk Of Bias In Non-randomized Studies of Interventions (ROBINS-I) [58], and the Newcastle–Ottawa Scale (NOS) for observational studies [59]. Two authors (AD & MB) will independently assess the risk of bias in each included study. Potential conflicts will be resolved through discussion.

### Measures of intervention effect

Where continuous scales of measurement are used to assess the effects of the intervention (e.g., mean number of servings of fruit and vegetable consumption, nutrition knowledge scores), the mean difference (MD) will be used, or the standardized mean difference (SMD) if different scales have been used. When considering studies with multiple intervention groups, we will try to combine all relevant experimental intervention groups into a single group to enable a single pairwise comparison. For considering cross-over studies, we will only use data from the first period. If the meta-analysis is not performed, the reason will be provided. At this stage, the findings will be summarized and discussed. Conclusions will also be formed based on the power of each of the studies. After summarizing the results and providing conclusions, the specific features associated with effective interventions will be identified.

### Dealing with missing data

Studies with missing data will also be included in the systematic review. Any further information required from the original author will be requested by written correspondence (e.g., emailing the corresponding author) and any relevant information obtained in this manner. Attrition rates, for example, drop-outs, losses to follow-up, and withdrawals will be investigated.

### Data synthesis

The data from each paper (e.g., study characteristics, context, participants, outcomes, and findings) will be used to build evidence tables of an overall description of included studies. Effect sizes (e.g., MD) will be presented along with 95% confidence intervals. If feasible and appropriate, effect sizes from primary studies will be used to perform random-effects meta-analyses. Since heterogeneity is expected a priori, we will estimate the pooled effect sizes using the random-effects model. The random-effects model assumes the study prevalence estimates follow a normal distribution, considering both within-study and between-study variation. Forest plots will be used to visualize the extent of heterogeneity among studies. We will quantify statistical heterogeneity by estimating the variance between studies using *I*^2^ statistic [60]. *I*^2^ ranges between 0% and 100% (with values of 0–25% and 75–100% taken to indicate low and considerable heterogeneity, respectively). We will also report Cochran *Q* test with a *P* value of 0.05 considered statistically significant (heterogeneity).

### Additional analyses

If sufficient data are available, we will conduct a subgroup analysis to explore possible heterogeneity sources (e.g., participants, interventions, and study quality). Heterogeneity among participants could be related to age (e.g., elderly, adults, children, and adolescents), sex (male versus female), geographic location (e.g., urban versus rural, country, or region), and socio-economic condition. Heterogeneity in interventions could be related to how the intervention is delivered, components of the intervention, the duration of the intervention, and settings (kindergarten, school, home, or community). Publication bias will be assessed by inspection of the funnel plots for asymmetry and with Egger’s test [61] and Begg’s test [62], with the results considered to indicate potential small study effects when *P* values are < 0.10.

### Software to be used

All analyses will be conducted using STATA (13.0; Stata Corporation, College Station, Texas, USA Stata).

### Quality assessment of included studies

Data extraction form also includes an overall grading of the evidence related to each of the main outcomes using the GRADE (Grades of Recommendation, Assessment, Development, and Evaluation) approach. It is the most flexible methodology for evaluating the evidence (downgrading, upgrading, handling indirect evidence, etc.) The GRADE approach defines the quality of a body of evidence as to the extent to which one can be confident that an estimate of effect or association is close to the true quantity of specific interest [63].

## Discussion

There is no doubt that diet quality has a major impact on health. A higher-quality diet has always been associated with better health outcomes [64, 65]. The relationship between diet and health is an undeniable cause-and-effect one [66]. This causal relationship has been proven through very detailed studies aimed at identifying causal pathways whereby specific nutrients affect health [67] and the high-quality intervention trials such as those incorporating the Mediterranean diet [68, 69]. Diet is currently among the leading preventable causes of mortality and disability in nearly all countries [70, 71]. Given the role of unhealthy nutrition in increasing the prevalence of non-communicable diseases [72], it is not unreasonable to expect an important step to prevent diseases and reduce mortality rates by correcting eating habits and food choices. However, the critical issue is that it is challenging to implement and maintain health-promoting behaviors, including healthy eating behaviors. This issue is significantly exacerbated in environments where low nutritional value and low-cost ready-to-eat foods are readily available and frequently advertised [73, 74].

Examining the available evidence and modeling based on practical and successful studies is considered the first step to facilitate policy improvement and design effective programs to promote healthy eating behaviors. On the other hand, an essential step for the final judgment on the types of interventions and their impact on promoting healthy eating behaviors is to conduct a systematic review of related studies [75]. Therefore, the present study is intended to collect articles related to interventions based on the social marketing approach to promoting healthy eating behaviors by searching for relevant scientific literature. After the final selection and extraction of papers, the components of the interventions, implementation methods, and their effectiveness will be identified and reported in a systematic review article to guide design interventions with the same purpose.

This systematic review will be restricted to articles in English due to functional reasons, and therefore could miss relevant papers published in other languages.

The review results can provide policymakers with the information needed to make decisions and plan to promote healthy eating behaviors and understand the factors influencing the programs’ implementation.

This review is intended for publication in a peer-reviewed journal. Analyses and scripts will be made publicly available. Any changes to the protocol will be documented as well.

## Supplementary Information

**Additional file 1.**

**Additional file 2.**

**Additional file 3.**

## Data Availability

Data sharing is not applicable to this article, as no datasets were generated or analyzed during the current study.

## Abbreviations

NCDs: Non-communicable diseases
WHO: World Health Organization
ISMA: International Social Marketing Association
ESMA: European Social Marketing Association
AASM: Australian Association of Social Marketing
PRISMA-P: Preferred Reporting Items for Systematic Review and Meta-Analysis Statement-Protocol Extension
RCT: Randomized controlled trial
DDS: Dietary Diversity Score
GRADE: Grades of Recommendation, Assessment, Development, and Evaluation
SMD: Standard mean difference
MD: Mean difference
