# Supplementary material for: Social marketing-based interventions to promote healthy nutrition behaviors: a systematic review protocol
Source: Syst Rev. 2021 Mar 11;10:75. doi: 10.1186/s13643-021-01625-5 (PMC7971101; doi:10.1186/s13643-021-01625-5)
Supplement: Supplementary file 2 — Additional file 2. [file 13643_2021_1625_MOESM2_ESM.docx]

# **Additional file 2**

**Draft search for main databases**

**Table1. Search strategies**

| **Database** | **Search terms** |
| --- | --- |
| **CENTRAL** | 1. [nutrition behavior] 2. [dietary behavior] 3. [eating behavior] 4. [dietary habit] 5. [food habit] 6. [milk consumption] 7. [dairy consumption] 8. [improvements in fruit and vegetable consumption] 9. [healthy food choices] 10. [nutrient-dense food choices] 11. [decrease unhealthy food] 12. [decrease low-nutrient energy-dense foods] 13. [healthy snacks] 14. [sweet snacks] 15. [salty snacks] 16. [eating main meals] 17. [eating breakfast] 18. [main meal eating] 19. [fast foods] 20. [water intake] 21. [sugary drinks] 22. behavior or behaviour:ti,ab,kw 23. {or #1-#22} 24. Social Marketing: ti,ab,kw 25. Intervent*:ti 26. {and #23, #24, #25} |
| **MEDLINE** | 1. nutrition behavior/ 2. dietary behavior/ 3. eating behavior/ 4. dietary habit/ 5. food habit/ 6. milk consumption/ 7. dairy consumption/ 8. improvements in fruit and vegetable consumption/ 9. healthy food choices/ 10. nutrient-dense food choices/ 11. decrease unhealthy food/ 12. decrease low-nutrient energy-dense foods/ 13. healthy snacks/ 14. sweet snacks/ 15. salty snacks/ 16. eating main meals/ 17. eating breakfast/ 18. main meal eating/ 19. fast foods/ 20. water intake/ 21. sugary drinks/ 22. (behavior or behaviour).tw. 23. or/1-22 24. (Social Marketing).tw. 25. Intervent*:tw 26. and/23,24, 25 |
| **EMBASE** | 1. nutrition behavior/ 2. dietary behavior/ 3. eating behavior/ 4. dietary habit/ 5. food habit/ 6. milk consumption/ 7. dairy consumption/ 8. improvements in fruit and vegetable consumption/ 9. healthy food choices/ 10. nutrient-dense food choices/ 11. decrease unhealthy food/ 12. decrease low-nutrient energy-dense foods/ 13. healthy snacks/ 14. sweet snacks/ 15. salty snacks/ 16. eating main meals/ 17. eating breakfast/ 18. main meal eating/ 19. fast foods/ 20. water intake/ 21. sugary drinks/ 22. (behavior or behaviour).tw. 23. or/1-22 24. (Social Marketing).tw. 25. Intervent*:tw 26. and/23,24, 25 |
| **ProQuest** | 1. nutrition behavior 2. dietary behavior 3. eating behavior 4. dietary habit 5. food habit 6. milk consumption 7. dairy consumption 8. improvements in fruit and vegetable consumption 9. healthy food choices 10. nutrient-dense food choices 11. decrease unhealthy food 12. decrease low-nutrient energy-dense foods 13. healthy snacks 14. sweet snacks 15. salty snacks 16. eating main meals 17. eating breakfast 18. main meal eating 19. fast foods 20. water intake 21. sugary drinks 22. (behavior or behaviour) in Citation and Abstract 23. or/1-22. in Citation and Abstract 24. randomized controlled trial 25. quasi experimental randomized control trial 26. cluster randomized trial 27. randomized 28. trial 29. groups 30. or/24-29. in Citation and Abstract 31. (Social Marketing) in Citation and Abstract 32. Intervent* in Citation and Abstract 33. and/23,30, 31, 32 |
| **Web of knowledge/All databases** | 1. nutrition behavior 2. dietary behavior 3. eating behavior 4. dietary habit 5. food habit 6. milk consumption 7. dairy consumption 8. improvements in fruit and vegetable consumption 9. healthy food choices 10. nutrient-dense food choices 11. decrease unhealthy food 12. decrease low-nutrient energy-dense foods 13. healthy snacks 14. sweet snacks 15. salty snacks 16. eating main meals 17. eating breakfast 18. main meal eating 19. fast foods 20. water intake 21. sugary drinks 22. (behavior or behaviour) 23. or/1-22.ti 24. (Social Marketing).ti 25. Intervent*:ti 26. and #23, #24, #25 |
