# Supplementary material for: Social marketing-based interventions to promote healthy nutrition behaviors: a systematic review protocol
Source: Syst Rev. 2021 Mar 11;10:75. doi: 10.1186/s13643-021-01625-5 (PMC7971101; doi:10.1186/s13643-021-01625-5)
Supplement: Supplementary file 3 — Additional file 3. [file 13643_2021_1625_MOESM3_ESM.docx]

# **Additional file 3**

**Draft of data extraction form**

**Characteristics of included studies** *(ordered by study ID)*

**Author, year (ref)**

| **Methods** |  | |
| --- | --- | --- |
| **Participants characteristics** |  | |
| **Intervention characteristics** |  | |
| **Outcomes results** |  | |
| ***Risk of bias (Grades of Recommendation, Assessment, Development and Evaluation (GRADE)) quality assessment tool for quantitative studies)*** | | |
| **Bias** | **Authors’ judgement** | **Support for judgement** |
| **Selection bias** |  |  |
| **performance bias** |  |  |
| **detection bias** |  |  |
| **attrition bias** |  |  |
| **reporting bias** |  |  |
| **other bias** |  |  |
| **Global rating for this papper** |  |  |
| **Overall assessment** |  |  |
